# Efficacy of Arthroscopic Treatment for Concurrent Medial Meniscus Posterior Horn and Lateral Meniscus Anterior Horn Injury: A Retrospective Single Center Study

**DOI:** 10.1111/os.12820

**Published:** 2020-11-27

**Authors:** Jun‐cheng Cui, Wen‐te Wu, Long Xin, Zhi‐wei Chen, Peng‐fei Lei

**Affiliations:** ^1^ Department of Orthopedics First Affiliated Hospital, University of South China Hengyang China; ^2^ Department of Orthopedics Xiangya Hospital Central South University Changsha China

**Keywords:** Arthroscopy, Injury, Meniscus, medial, lateral, Repair

## Abstract

**Objective:**

To investigate the effectiveness of arthroscopic surgery for combined tears of the posterior horn of the medial meniscus (PHMM) and the anterior horn of the lateral meniscus (AHLM).

**Methods:**

Between September 2009 and December 2013, a retrospective investigation was performed on 48 patients (48 knees) with combined tears of PHMM and AHLM who underwent arthroscopic surgery. All patients underwent magnetic resonance imaging (MRI) diagnosis in the outpatient department. After admission, other basic examinations were performed. PHMM was treated with partial meniscectomy and AHLM was treated with external–internal suture or partial meniscectomy. Curative effect was evaluated according to Lysholm score and IKDC score. The differences of the functions as well as Lysholm and IKDC scores pre‐ and postoperatively were compared, while the diagnostic accuracy of MRI and arthroscopy for PHMM and AHLM were compared.

**Results:**

Seven patients with combined tears of PHMM and AHLM were misdiagnosed during MRI examination when arthroscopy was used as the gold standard. All patients were followed up for an average of 34.58 months (range 10–52 months) and all incisions healed by first intention with no obvious complication. The preoperative and postoperative Lysholm scores were 47.22 ± 2.77 and 87.36 ± 5.45, respectively. The IKDC scores are 54.73 ± 4.65 preoperatively and 89.62 ± 3.71 postoperatively. The positive rates of the diagnosis through MRI and arthroscopic surgery is 85.42% and 100%, respectively. At the last follow‐up, the patients had no pain, weakness, and instability, and tenderness in medial and lateral joint space disappeared. Mcmurray test was weakly positive in four patients. Excellent outcome was achieved in 39 cases, and a good outcome was achieved in five cases; the good to excellent rate was 91.67%.

**Conclusion:**

MRI examination of combined tears of PHMM and AHLM may result in misdiagnosis. Arthroscopic primary repair seemed to be an effective surgical option for treatment of combined tears of PHMM and AHLM.

## Graphical abstract

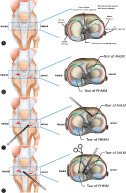

Schematic diagram of arthroscopy of meniscus injury following surgery; (A) The anatomic structure of the knee; (B) AHLM and PHMM injuries of the meniscus; (C) Description of the performed meniscus injury with suture anterior horn of the lateral meniscus; (D) Description of the performed meniscus injury with removal of the incomplete bacterial horn of the medial meniscus.

## Introduction

Meniscal tears are the most common knee injury in the clinic and usually occur during sports^1^. Meniscus tears can be divided into acute injury and chronic injury, which is closely related to age, occupation, and activity^2^. Meniscal tears are also one of the most frequent indications for knee arthroscopy^3^ and they can be vertical or horizontal and can also be complex, combining two or more than two injury types. Meniscus injuries are very common and can affect the lives of people or the careers of athletes, which brings a huge economic burden to society. As people's lifestyles change and the number of people exercising increases, the impact of such injuries will further increase. Therefore, it is very important for the accurate diagnosis and surgical treatment of meniscus injury and postoperative rehabilitation.

MRI serves as a useful screening tool in patients with an acute knee injury, often allowing orthopaedic surgeons to identify those who would benefit from surgery^2^, ^4^, ^5^. However, the sensitivity of MRI for the detection of meniscal tears is not yet 100%, and athletes have occasionally returned to activity with undiagnosed meniscal tears on the basis of a normal MRI examination^6^. A meta‐analysis of the diagnostic accuracy of magnetic resonance imaging (MRI) for meniscal tears of the knee revealed a pooled sensitivity of 89% (95% CI 83–94%) for medial meniscal tears and 78% (95% CI 66–87%) for lateral meniscal tears^7^, indicating that MRI may miss close to 20% of cases of lateral meniscal tears. Smet and Graf analyzed the MRI reports and surgical records of 400 patients who had both an MRI examination and arthroscopy of the knee^8^ and found that subtle peripheral tears in the posterior horn of the lateral meniscus were very difficult to detect on MRI. Laundre *et al*. studied 120 patients (<40 years old) who underwent arthroscopic anterior cruciate ligament (ACL) reconstruction within 6 weeks after MRI to identify MRI‐missed meniscal tears and found that the majority (19/28) of missed tears involved the posterior horn of the lateral meniscus^9^. A recent systemic review of seven prospective studies showed that B‐mode ultrasound used to detect meniscal tears showed a pooled sensitivity of 88.80% (95% CI: 82.83–92.87)^10^.

Posterior horn meniscus tears may also be accompanied by tears on anterior horn of the lateral meniscus. The combined anterior horn meniscus tears could be misdiagnosed preoperatively on MRI or clinically, causing delay in surgery and seriously affecting the patient's knee^11^. Few reports are currently available on the associated tears in the posterior horn of the medial meniscus and the anterior horn of the lateral meniscus.

In the current retrospective study, we analyzed: (i) the diagnostic accuracy of MRI for PHMM and AHLM; (ii) the effectiveness of arthroscopic treatment for knee meniscus injury patients who had combined medial meniscus posterior horn and lateral meniscus anterior horn tears; and (iii) the prognosis of patients with PHMM and AHLM followed arthroscopic treatment.

## Patients and Methods

### *Inclusion Criteria*

Patients with PHMM and AHLM were treated with arthroscopic surgery according to the standard, and the preoperative and postoperative Lycholm score and IKDC score were calculated respectively. Inclusion criteria: (i) patients with combined injury of PHMM and AHLM confirmed by clinical MRI or arthroscopy; (ii) patients with no special treatment before admission. Exclusion criteria: (i) patients with severe bone and joint degeneration; (ii) patients with severe articular cartilage and ligament injury; (iii) patients with surgical contraindications; (iv) patients with less than 1 year follow‐up; (v) patients with bilateral knee injuries or severe synovitis.

### *Patients*

The study protocol was approved by the local ethics committee of the authors' affiliated institution. Patient informed consent was not required because of the retrospective nature of the study and patient data were anonymized in the report. Between September 2009 and December 2013, the medical records for demographic, radiological, and surgical data of patients with combined injury of PHMM and AHLM who underwent arthroscopic surgery were retrospectively reviewed. All patients underwent MRI diagnosis in the outpatient department. After admission, blood routine, liver and kidney function, electrolyte, blood type, pre‐transfusion examination, chest DR, and other basic examinations were performed. Knee arthroscopic findings were used to determine: the distribution of injuries (anterior horn, body, or posterior horn; tears involving one, two, or three segments); configuration of tears (complex, bucket handle, displaced flap, oblique, radial, longitudinal peripheral, partial thickness, or split type); surgical treatment of meniscal tears (meniscal repair or resection or partial meniscectomy). Injuries older than a year at the time of arthroscopic treatment were considered as chronic injuries. (Table 1.)

**TABLE 1:** General clinical data of all patients

	Number of case(n,%)
	48 (100.00)
Gender	
Female	29 (60.42)
Male	19 (39.58)
Age (years)	49.56 (ranging: 30–65)
Affected side	
Left	17 (35.42)
Right	31 (64.58)
Duration of disease (months)	29.47 (3–107)
Time of follow‐up (months)	34.58 (10–52)
Outcome	19 18
Excellent	34 (70.84)
Good	10 (20.83)
Fair	4 (8.33)

### *Surgical Technique*

#### *Anesthesia and Surgical Position*

Before surgery, the patient was placed in the supine position with continuous epidural anesthesia combined with subarachnoid block anesthesia. A tourniquet was placed around the thigh applying a pressure of 60 kPa. All surgical procedures were performed by the senior author with more than 10 years' experience.

#### *Surgical Approach and Exposure*

Subpatellar anterolateral and anteromedial approaches were taken. The anterolateral approach was used. Puncture was performed at 1 cm lateral patellar tendon and above anterior horn of lateral meniscus. Then, arthroscopy was placed, 0.9% sodium chloride injection was injected under pressure and assisted by a probe hook.

#### *Operation Technique*

**Fig 1:**
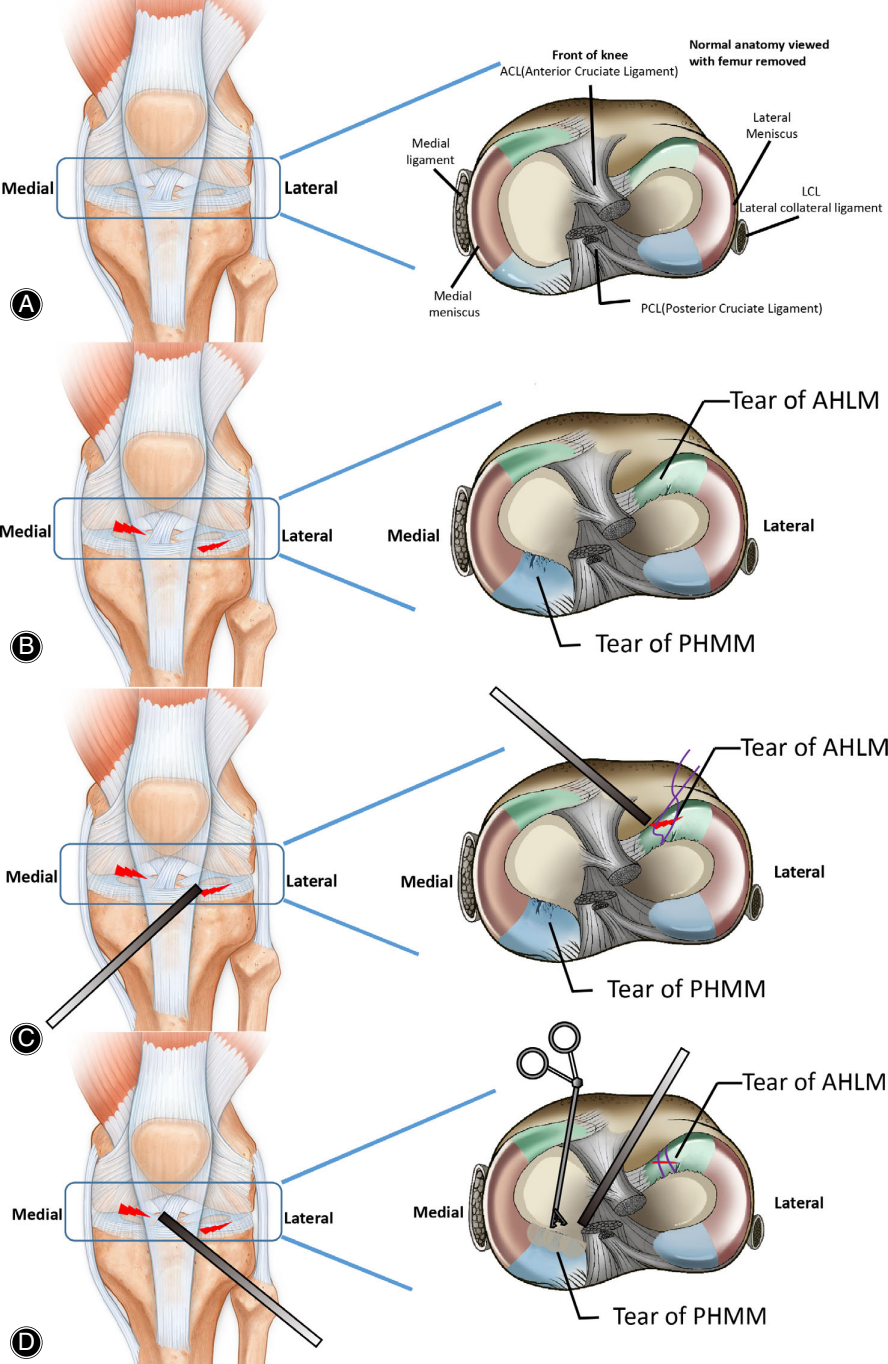
Schematic of arthroscopy of meniscus injury following surgery; (A) The anatomic structure of the knee; (B) AHLM and PHMM injuries of the meniscus; (C) Description of the performed meniscus injury with suture anterior horn of the lateral meniscus; (D) Description of the performed meniscus injury with removal of the incomplete bacterial horn of the medial meniscus.

The surgical procedures are shown in Fig. 1. The intraoperative status of the menisci and cartilage was documented by orderly examination of the upper patellar capsule, patellar joint surface, the femoral condyle trochlear, meniscus, cruciate ligament, and the medial and lateral compartment. The location, extent, and shape of meniscus injury were assessed. Synovial hyperplasia in the articular cavity was observed under the microscope and excised with a chipper. For the posterior horn injury of the medial meniscus, partial resection was performed to the normal meniscus tissue with a basket clamp and a planer (for radial fracture, the free part of the meniscus tissue was removed) and then the meniscus slope was trimmed with an electrocoagulation gasification knife. Care was taken not to damage the meniscus and the cruciate ligament. Partial meniscectomy was undertaken based on the type and extent of the tear in the posterior horn of the medial meniscus. The radial fissure of the lateral meniscus was treated in the same manner as posterior horn injury in the medial meniscus. And to maintain the meniscus area and delay the occurrence of osteoarthritis, longitudinal tear was not resected and repaired by external–internal suture with No. 2 suture line. Because of the small incision of knee arthroscopic surgery, the incision was not sutured after operation. After skin edge alignment, the lower limbs were directly compressed and elastically bandaged from the middle of the foot up to the middle and lower thigh.

### *Rehabilitation Program*

The involved limb was kept elevated with a soft pillow pad for 2 to 3 days. Rehabilitation exercise was provided where appropriate. Quadriceps stretches and straight leg raising training were started on postoperative day (POD) 1 or 2. Dressing change was done and the elastic bandage was removed on POD 1. Patients were asked to carry out partial weight‐bearing activities on walking sticks and started knee flexion exercise 1 week postoperatively. Patients started full weight‐bearing activities by week 8. Outcomes were evaluated using the Lysholm score scale^12^.

### *Follow‐up and Efficacy Analysis*

#### *Lysholm Score*

Lysholm knee scale is a subjective scoring system for patients with knee ligament or meniscal injuries^13^, ^14^. This systematic score were used to compare the knee function of the patient before and at 6, 12, 24, and 48 months after surgery. In the Lysholm score, an overall score of 0 to 100 is calculated and graded based on eight domains: limp, locking, pain, stair climbing, support, instability, swelling, and squatting. The efficacy was evaluated according to Lysholm score. A score of 95 to 100 is considered excellent, 84 to 94 is good, 65 to 83 is fair, and <65 is poor.

#### *International Knee Documentation Committee (IKDC)*

IKDC form is an assessment tool used to detect improvement or deterioration in symptoms, function, and sports activities for patients with a variety of knee conditions, including ligament injuries, meniscal injuries, articular cartilage lesions, and patellofemoral pain^14^. It was also used to compare the knee function of the patient before and at 6, 12, 24, and 48 months after surgery. The IKDC is a patient‐completed tool, which contains sections on knee symptoms (seven items), function (two items), and sports activities (two items). A score of 95 to 100 is considered excellent, 84 to 94 is good, 65 to 83 is fair, and <65 is poor.

### *Statistical Analysis*

Statistical analysis was conducted using SPSS Statistics software version 19.0 (IBM, Armonk, NY). Two‐sided paired Student's *t* test with unequal variance and Fisher's exact test were used to analyze preoperative and postoperative continuous and categorical variables, respectively. Statistical significance was defined as a *P* value of <0.05. The data are presented as the mean with range or the number and percentage.

## Results

### *Patient Demographics*

General clinical data of all patients was shown in Fig. 1. Forty‐eight cases (48 knees) had both posterior horn injury of the medial meniscus and anterior horn injury of the lateral meniscus. Their mean age was 49.56 years (range 30 to 65 years) and 60.42% of them were female. The left knee was involved in 17 (35.42%) cases and the right knee was injured in 31 (64.58%) cases. Sprain was identified as the cause of knee injury in three cases; no obvious trigger was found in the remaining cases. The median duration of the disease was 29.47 months (range 3 months to 107 months).

Admission examination revealed a median angle of 98.7° (range, 85–120°) for knee flexion and a median angle of −2.5° (range, − 8 to 4°) for knee extension. Arthrohydrops were present in five cases. Forty‐three cases had longitudinal fissure and five cases had radial fissure. Forty‐two cases had posterior horn injury of the medial meniscus with concurrent anterior horn injury of the lateral meniscus injury, and six cases with posterior horn injury of the medial meniscus injury had no obvious abnormality in the lateral meniscus in preoperative MRI and X‐ray. For the posterior horn injury of the medial meniscus, partial resection was performed to the normal meniscus tissue with a basket clamp and a planer (for radial fracture, the free part of the meniscus tissue was removed) and then the meniscus slope was trimmed with an electrocoagulation gasification knife. Longitudinal tear was not resected and repaired by external–internal suture with No. 2 suture line. In addition, two cases had meniscus cyst, with popliteal fossa cyst in one case. The cyst communicated with the joint cavity through the meniscus cleft, which was located at the edge of the posterior horn of the medial meniscus. No patients had injuries of the cruciate ligaments and collateral ligaments.

The posterior horn injury of the medial meniscus was located in the white zone. Forty cases had radial crack, six cases had lamination, and two cases had radial crack and lamination. MRI failed to detect anterior horn injury of lateral meniscus in six (16.7%) cases, all of which were longitudinal fissure in the red zone. As a result, the accuracy rate of diagnosis by MRI is 83.3%. The remaining 42 cases were located in the red zone (19 cases) or the red‐white zone.

### *Surgical Outcomes*

#### *Lysholm Score*

The mean Lysholm score at the final follow‐up improved significantly from 47.22 ± 2.77 preoperatively to 87.36 ± 5.45. The Lysholm Score significantly improved postoperatively compared with that preoperatively in PHMM and AHLM patients. According to the improved Lysholm score scale, the surgical outcome was excellent in 34 cases, good in 10 cases, and fair in four cases, with a good to excellent rate of 91.67%.

#### *IKDC Score*

The mean IKDC score improved significantly from 54.73 ± 4.65 preoperatively to 89.62 ± 3.71 at the last follow‐up. The IKDC score also significantly improved postoperatively compared with that preoperatively in patients and the surgical outcome was the same as the Lysholm score. (Table 2.)

**TABLE 2:** Preoperative and postoperative Lysholm and IKDC scores of the patients

	Preoperative score	Postoperative score	*P*‐valve
Lysholm score	47.22 ± 2.77	87.36 ± 5.45	*P* < 0.001
IKDC score	54.73 ± 4.65	89.62 ± 3.71	*P* < 0.001

#### *Complication*

**Fig 2:**
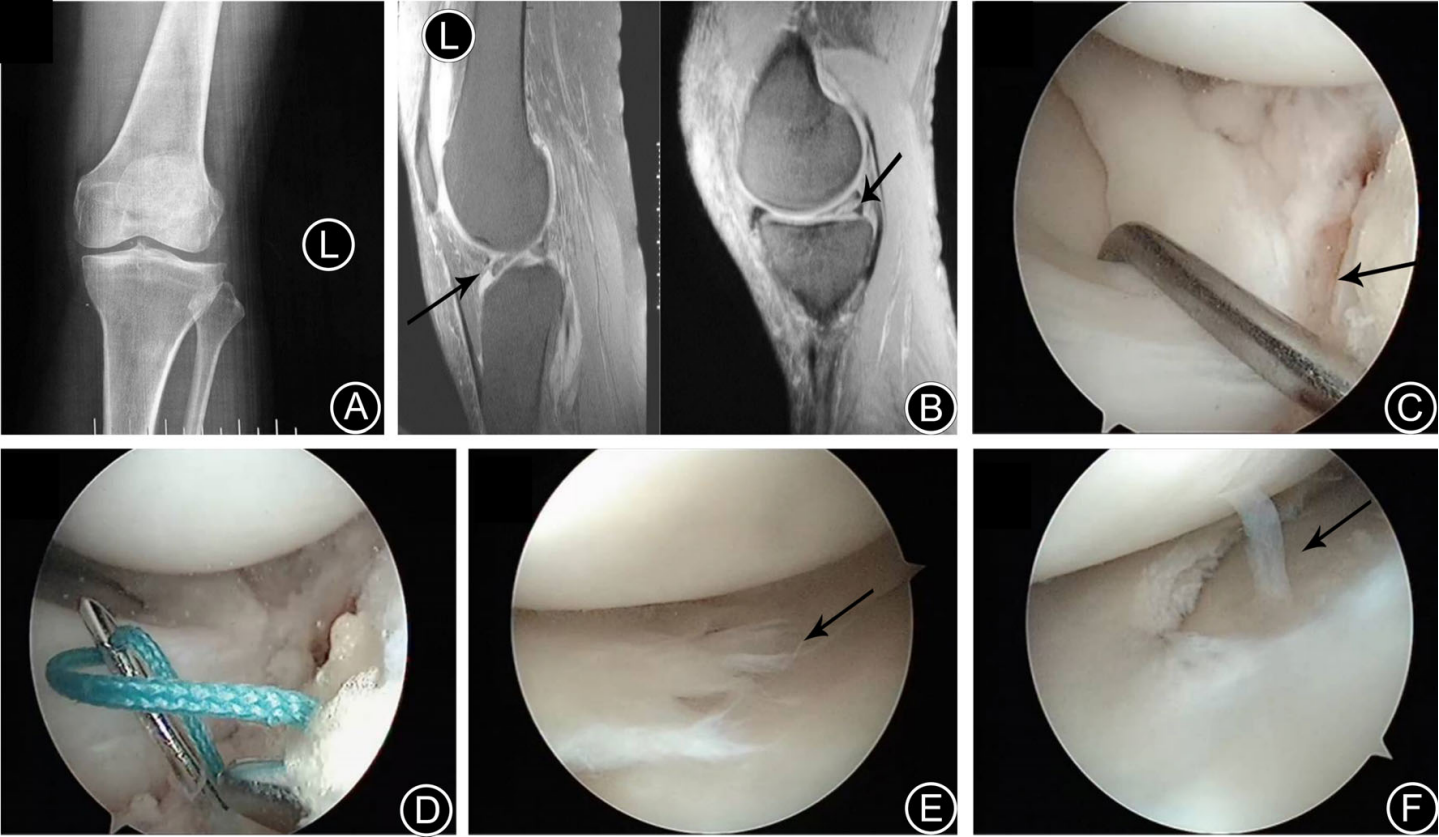
A 53‐year‐old female patient with combined injury of posterior horn medial meniscus and anterior horn lateral meniscus in the left knee. (A) Anteroposterior X‐ray film before operation; (B) Sagittal MRI before operation; black arrow indicates longitudinal tears of anterior horn lateral meniscus and horizontal tears of posterior horn medial meniscus; (C) Longitudinal tears of anterior horn lateral meniscus under arthroscope (arrow); (D) The anterior horn lateral meniscus after suturing; (E) Horizontal tears of posterior horn medial meniscus under arthroscope (arrow); (F) The posterior horn medial meniscus after menisectomy.

**Fig 3:**
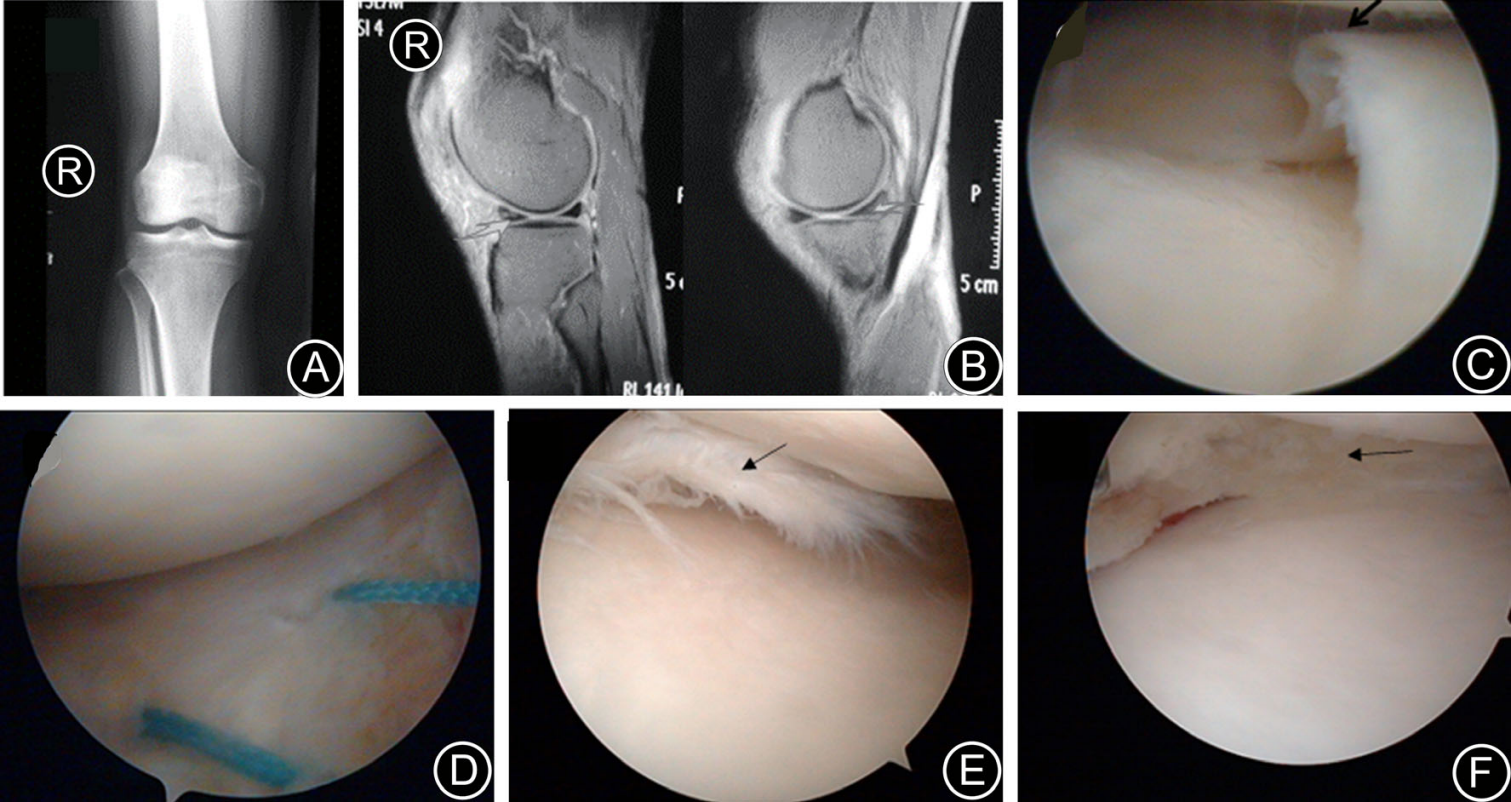
A 47‐year‐old male patient with combined injury of posterior horn medial meniscus and anterior horn lateral meniscus in the right knee. (A) Anteroposterior X‐ray film before operation; (B) Sagittal MRI before operation; black arrow indicates longitudinal tears of anterior horn lateral meniscus and horizontal tears of posterior horn medial meniscus; (C) Longitudinal tears of anterior horn lateral meniscus under arthroscope (arrow); (D) The anterior horn lateral meniscus after suturing; (E) Horizontal tears of posterior horn medial meniscus under arthroscope (arrow); (F) The posterior horn medial meniscus after menisectomy.

All the study patients underwent partial or subtotal meniscectomy. One patient with popliteal cyst underwent open surgical excision of the cyst. Stage I healing was achieved in all the wounds. No nerve injury, joint or wound infection, joint stiffness, or other complications were observed. The patients were followed up for a median mean duration of 34.5 months (range 10 − 52 months). At the final follow‐up, no patients had knee joint instability or weakness, and knee joint or lateral gap tenderness. The McMurray sign was weakly positive in four cases and negative in the remaining cases. Four cases had slight pain in the medial space behind the knee and 44 cases reported no knee joint pain. The cysts were absorbed and disappeared in two cases of meniscus cyst. Knee joint activity was not limited, and the median angle of knee flexion was 123.6° (range 110 ~ 145°) and that of knee extension was 3.8° (range 0 ~ 6°). A representative case is shown in Fig. 2 of a 56‐year‐old female patient with combined injury of posterior horn medial meniscus and anterior horn lateral meniscus in the left knee. Another representative case is shown in Fig. 3 of a 47‐year‐old male patient with the same disease in the right knee.

## Discussion

As an important structure in the knee joint, meniscus is an intra‐articular filler, which can increase the contact surface between femur and tibia, thus improving the stability of the knee joint, effectively conducting the pressure load, and alleviating shock^15^. The majority of meniscal injuries in young adults are caused by direct or indirect violence. Meniscus injuries in middle‐aged and elderly patients are commonly related to meniscus degeneration^1^. In the course of arthroscopic treatment of knee meniscus injury, we found that some patients with combined partial medial meniscus posterior horn injury and lateral meniscus anterior horn injury were easily missed before operation. For such patients, not identifying and treating them in time will seriously affect the recovery of knee function. Currently, there are rare reports on such injuries. For this reason, we reviewed the clinical data of these 48 patients and summarized the results. The mechanism of injury and the effect of arthroscopic treatment may provide a reference for clinical diagnosis and treatment for concurrent medial meniscus posterior horn and lateral meniscus anterior horn injury.

The current study reports the successful treatment of 48 cases of combined posterior horn injury of the medial meniscus and anterior horn injury of the lateral meniscus. It should be noted that most of these combined injuries are caused by chronic strain. Meniscus injury is the most common injury of the knee joint, which usually occurs in contact movement^16^. Nagura *et al*.^17^ showed that the pressure in the posterior knee in high flexion is 58.3% − 67.8% higher than that of walking, and persistent high extension could easily injure the meniscus. In Asian countries, people often kneel down, squat, and sit with legs crossed, resulting in knee flexion >120°^18^ and as high as 157 to 165°^19^, ^20^. Only three cases in our series had a clear history of ankle sprain while the cause of meniscus tear in the majority of the cohort is unknown. We speculate that squatting or sitting with legs crossed might be a contributor to meniscus injury, but clinical evidence is required to support this hypothesis.

In medial meniscus posterior horn injury combined with anterior meniscus injury, the injury of the posterior horn of the medial meniscus may cause biomechanical changes of the knee joint, and result in injury of the lateral meniscus anterior horn.^21^ Huang *et al*.^22^ showed that after unilateral meniscus resection, the axial load of the contralateral meniscus and the maximum equivalent stress on the surface of the meniscus all increased. Allaire *et al*.^23^ studied the biomechanical changes after posterior horn injury of the medial meniscus and showed that medial meniscus posterior horn injury had effects similar to meniscectomy in terms of knee joint stress and motion mechanics, not only causing the medial meniscus maximum response but also an increase in the maximum stress of the lateral meniscus as well as an increase in force and contact area. At the same time, there are a series of biomechanical changes such as tibial forward and outward movement, increase of the external rotation angle of the tibia, and the varus of the knee joint^24^.

The posterior 1/3 of the medial meniscus limits knee external rotation.^25^ Allaire *et al*.^23^ showed that after total meniscectomy, the external rotation angle of the tibia increased by 4.45°, and external displacement increased by 0.80 mm. When the tibia is rotated, the posterior horn of the medial meniscus is closely attached to the internal condyle of the femur and the anterior horn of the lateral meniscus is attached to the external condyle of the femur. In meniscus posterior horn injury, the tibial external rotation angle will augment this effect, and also increase the tibial shift. The anterior horn of the lateral meniscus and femur condyle are tightly attached; as a result, relative displacement range increases, resulting in tear. The fibers on the lateral 2/3 of the meniscus are circularly distributed. After repeated rubbing in the horizontal direction of the lateral condyle of the femur, the fiber bundles are easily split and form longitudinal fractures, which is consistent with the main types of longitudinal injuries of the lateral meniscus anterior horn.

In the current series, partial meniscectomy was used for medial meniscus posterior horn tear in the white zone, so functioning meniscus tissues were preserved as much as possible and only damaged tissues or portioning showing unstable mechanical properties were removed. Efforts should be made to preserve a concave meniscus with a smooth edge, and the surrounding fibrous bands. The biomechanical changes caused by loss of the posterior horn of the medial meniscus should be minimized to lessen degerenntative changes in the knee joint.

At present, the accuracy of MRI diagnosis of meniscus injury is 80% to 100%, but few small tears are easily missed^26^. Choi *et al* ^27^ and Shepard *et al*.^28^ believed that anterior meniscus tear was difficult to diagnose on MRI. De Smet *et al*.^29^ showed that the sensitivity of MRI in the diagnosis of lateral meniscus tear was only 81%. A meta‐analysis by Oei *et al*.^30^ revealed that the sensitivity of MRI was only 79.3% in the diagnosis of lateral meniscus. All of these studies suggest that some cases of meniscus tear may be missed by preoperative MRI. Anterior horn injury of the lateral meniscus was missed in six patients in our series by preoperative MRI. Preoperative knee joint examination should be performed in detail, and high‐resolution MRI should be used to increase the sensitivity of preoperative MRI, which will aid intraoperative arthroscopic surgery in fine exploration of the suprapatellar bursa, patellofemoral joint, femoral trochlea, meniscus, cruciate ligament, and medial and lateral compartment structures when the posterior horn of the medial meniscus is injured. The meniscus has a certain self‐healing ability, and Petersen *et al*.^31^ show that the healing rate of simple meniscus tear is 50–75% with suturing in the red zone and the red‐white zone. The anterior horn injury of the lateral meniscus in our series was located in the red zone and the red‐white zone. The anterior horn was not resected and was repaired by suture. The knee joint activity of the patients recovered well after operation, with a good to excellent rate of 91.67%.

The conditions that should be noted during surgery are as follows: (i) Preoperative diagnosis should not rely solely on MRI imaging. During the operation, a thorough and careful examination should be made under arthroscopy. When the injury of the posterior horn of the medial meniscus is found, the injury of the anterior horn of the lateral meniscus should be assessed vigilantly to avoid missed diagnosis. (ii) For medial meniscus posterior horn injury, the functional part should be retained to the maximum extent, except the damaged and unstable part of mechanical properties. At the same time, a concave and smooth meniscus margin and a fibrous ring around the meniscus should be preserved as far as possible to reduce the biomechanical changes of the knee joint caused by the defect of the posterior horn of the medial meniscus and to slow down the degeneration of the knee joint. (iii) When the injury of anterior horn of lateral meniscus is located in red zone and red‐white zone, the healing rate is high after suture repair. Therefore, the outer–inner suture method was used to repair the injury of lateral meniscus in red zone and red‐white zone. However, because most of these combined injuries were caused by chronic strain, it is necessary to freshen the cleft edge with a planer first, and then sew it up to promote meniscus healing.

In conclusion, combined injury of medial meniscus posterior horn and lateral meniscus anterior horn injury may be missed by preoperative MRI. Preoperative examination and careful examination under arthroscopy can improve the diagnostic rate of joint injury and lead to a satisfactory outcome.

## Competing Interests

The authors declare that they have no competing interests.

## Funding

This work was supported by the Natural Science Foundation of Hunan Province, China (Grant. No. 2018JJ3468). The Natural Science Foundation of Hunan Province, China (Grant No. 2018JJ3844 and 2019JJ40499). The Scientific Research Project of Health and Family Planning Commission of Hunan Province, China (Grant No. B2019188). The Young Science Foundation of Xiangya Hospital Central South University (Grant No. 2017Q07). The Postdoctoral Research Program of Xiangya Hospital Central South University (Grant No. 223551). National key research and development project (2016YFC1100605 and 2018YFB1105504), Natural Science Foundation of China (Grant No. 81672656).

## Authors' Contributions

Juncheng Cui analyzed and interpreted the patient data regarding sports injuries and surgery. Wente Wu performed statistical analysis and surgery, and was a major contributor in writing the manuscript. All authors read and approved the final manuscript.

## Availability of data and materials

The data in this article is kept by the author. If necessary, you can contact the author to receive this data. Correspondence author: Chen Zhiwei, Department of Orthopedics,The First Affiliated Hospital of University of South China, No 69 Chuanshan Road, Hengyang,Hunan 421001, China (e‐mail: czw9915@sina.com); Lei Pengfei*, Department of Orthopedic Surgery, Xiangya Hospital Central South University, 87 Xiangya Road, Changsha, Hunan, China, 410007. E‐mail: pengfeilei@csu.edu.cn Tel: 073189753006, Fax: 0731–89753706.
